# Myocardial Injury in Critically Ill Children: A Case Control Study

**DOI:** 10.1155/2014/919150

**Published:** 2014-02-11

**Authors:** Basheir Hassan, Saed Morsy, Ahmed Siam, Al Shaymaa Ali, Mohamed Abdo, Mona Al Shafie, Ahmad Hassaneen

**Affiliations:** ^1^Pediatrics Department, Faculty of Medicine, Zagazig University, Zagazig 44519, Egypt; ^2^Clinical Pathology Department, Faculty of Medicine, Zagazig University, Zagazig 44519, Egypt

## Abstract

*Objectives*. The aim of this study was to investigate the occurrence of myocardial injury in critically ill children through assessment of cardiac troponin T levels and whether levels are associated with disease severity and myocardial dysfunction measured by echocardiography. *Methods*. Over a 6-month period, this case control study included 50 patients admitted to Pediatric Intensive Care Unit of Zagazig University Children's Hospital. Twenty-five healthy children were included as a control group. Demographic and clinical data including the pediatric index of mortality II score were recorded. Echocardiographic examination was done and level of cardiac troponin T was measured using Elecsys Troponin T STAT Immunoassay. *Results*. Cardiac troponin T levels were significantly higher in critically ill in comparison to healthy children (median 22 (18–28) pg/mL versus 10 (10-10) pg/mL, *P* < 0.05). Cardiac troponin T levels correlated positively with duration of ventilation as well as with disease severity and correlated negatively with left ventricular fractional shortening. Moreover, cardiac troponin T levels were significantly higher in nonsurvivors when compared to survivors (median 34.5 (27.5–41.5) pg/mL versus 20 (18–24) pg/mL, *P* < 0.05). *Conclusion*. In critically ill children, cardiac troponin T levels were elevated and were associated with duration of ventilation and disease severity.

## 1. Introduction

Cardiac troponins I and T are regulatory proteins that control the calcium-mediated interaction of actin and myosin, producing myocardial contraction [1]. Since troponins do not occur in extracellular space, their appearance in serum is sensitive and specific marker of myocardium damage [2]. Cardiac specific troponins T and I have been established as the gold standard biochemical markers for myocardial necrosis [3].

An unexpectedly high incidence of clinically unrecognized myocardial injury, assessed by elevated cardiac troponin I levels, has previously been reported in critically ill adult patients [4].

Elevated cardiac troponins levels have been detected in children critically ill with congenital heart disease before and after cardiac surgery [5]. In patients without congenital heart disease raised cardiac troponin levels have been found in pediatric intensive care unit (PICU) admissions with severe respiratory syncytial virus infection [6] and with meningococcal and other forms of septicemia [7, 8].

To our knowledge, there is no published data concerning myocardial injury in PICUs in our country. So, the aim of this study was to evaluate myocardial injury in critically ill children in our PICU and to assess relation between cardiac troponin T (cTnT) levels and disease severity as well with myocardial dysfunction measured by echocardiography.

## 2. Patients and Methods

The study was conducted prospectively as a case control study within the Zagazig University Children's Hospital PICU, from June 2012 through December 2012. Patients were included with age from >1 month to 12 years, of different admission etiologies other than congenital or acquired heart diseases. Patients with abnormal renal function were excluded from the study.

Twenty-five age and sex matched healthy children with no history of critical illness or chronic disease coming for elective specific general surgery and having routine outpatient phlebotomy were included as a control group.

Informed consent was obtained prior to inclusion in the study from the children's guardians. The study protocol was approved by the Pediatric Committee in Zagazig University.

Demographic and clinical data were recorded, including age, diagnosis, pediatric index of mortality II (PIM II) score, and outcome. Laboratory investigations included complete blood count, C-reactive protein, arterial blood gases, renal function, and measurement of serum cTnT level.

### 2.1. Estimation of Cardiac Troponin T Level

Samples were obtained on admission to PICU. Samples were spun, separated, and frozen at −20°C until batch analysis was performed. The assay was done with an Elecsys 1010 System Analyzer using the Elecsys Troponin T STAT Immunoassay (Roche Diagnostics GmbH, Mannheim, Germany) [9].

### 2.2. Echocardiographic Examination

All patients and controls underwent echocardiographic examination to assess the cardiac functions on the same day where the blood samples for cTnT were obtained. Echocardiography was performed at the bedside using GE vivid-7 multipurpose system with different probe sizes. Each patient was examined according to the recommendations of the American Society of Echocardiography [10]. Echocardiographic examination included M-mode, two-dimensional, and Doppler echocardiography. The left ventricular end systolic diameter, left ventricular end diastolic diameter, ejection fraction, and fractional shortening were measured using M-mode echocardiography in the left parasternal view.

### 2.3. Statistical Analysis

Data were analyzed using Statistical Package for Social Sciences (SPSS) release 16. Nonparametric value represented as median and interquartile range (IQL) and the median of two groups was tested by Mann-Whitney test. Qualitative data are represented by frequency and relative percentage and chi-square test was used for testing association of qualitative data. Correlations were performed using Spearman's rank correlation. In all analyses, *P* value of <0.05 was considered statistically significant.

## 3. Results

During the study period, 50 critically ill children were included in the study. Details of the baseline characteristics are given in Table 1.

There was highly significant increase in the serum level of cTnT in the critically ill children in comparison to the control group (median 22 (18–28) pg/mL versus 10 (10-10) pg/mL, *P* < 0.05; Table 1). Also, critically ill children had a significantly lower fractional shortening (*P* < 0.05).

During their ICU stay, 26 (52%) patients required mechanical ventilation. Overall mortality was 32% (16 of 50 patients). There were highly significant positive correlations between the level of cTnT and the duration of ventilation as well with PIM II score and highly significant negative correlation with the fractional shortening (Table 2).

As regards relation between cTnT and outcome, nonsurvived children had higher cTnT levels when compared with survivors (median 34.5 (27.5–41.5) pg/mL versus 20 (18–24) pg/mL, *P* < 0.05; Table 3).

## 4. Discussion

Acute severe myocardial dysfunction remains a significant cause of mortality and morbidity in children requiring intensive care [11]. In this study, we aimed to evaluate myocardial injury in our PICU patients using cTnT assay.

This prospective study showed that critically ill children had significantly higher level of cTnT when compared with healthy children. The same finding was documented in Clark et al. study [12] which investigated cTnT levels in critically ill ventilated infants. Other studies describe elevated cardiac troponin I among different groups of critically ill children as children with sepsis [7], meningococcemia [8], and RSV infections [6].

In ICU patients, physiologic stresses can occur in the form of either increased myocardial oxygen demands (e.g., fever, tachycardia) or decreased myocardial oxygen delivery (e.g., anemia, hypotension, and hypoxemia) resulting in cardiac dysfunction, cardiac injury, or both [13]. This potential for an imbalance between oxygen supply and demand and the known propensity of critically ill patients to develop acute thrombosis may explain the increase in the risk of myocardial injury [14, 15].

In our study, cTnT levels were positively correlated with the duration of ventilation. Trevisanuto et al. [16] found significant positive correlation between the levels of cTnT and the duration of ventilation in neonates. However, Clark et al. [12] and Eisenhut et al. [6] found no relation between cTnT levels and the duration of ventilation in infants.

In the current study, there were highly significant positive correlations between the level of cTnT and PIM II score as a measure of disease severity. Clark et al. [12] and Fenton et al. [17] documented this result in their studies.

As regards echocardiographic data, the current study revealed a highly significant negative correlation between levels of cTnT and left ventricular fractional shortening. In preterm neonates, El-Khuffash et al. [18] stated that there was a significant inverse correlation between cTnT and echocardiographic markers of myocardial function.

Results of our study also showed that there was highly significant difference between levels of cTnT in cases who died and those who survived, being higher in nonsurvivors. This finding was in agreement with that of Spies et al. study [19] in which cTnT as associated with an increased mortality rate. Also, in adult patients, Quenot et al. [20] stated that myocardial injury was shown to be an independent determinant of in-hospital mortality.

## 5. Conclusion

Myocardial injury, detected with elevated cTnT levels, occurs in critically ill children. CTnT levels correlated significantly with disease severity and left ventricular systolic functions. An association between mortality and elevated cTnT levels was found.

## Figures and Tables

**Table 1:** Demographic, clinical, and laboratory data of studied population.

Variable	Cases (n = 50)	Controls (n = 25)	*P* value
Age (months), median (IQL)	20 (9–28)	19 (9–29)	0.676
Gender, *n* (M/F)	29/21	13/12	0.622
Diagnosis			
Bronchiolitis	12	NA	NA
Bacterial lower RTI	6		
Sepsis	9		
CNS diseases	8		
Major abdominal surgery	3		
Hepatic diseases	5		
Others	7		
Mechanical ventilation, *n*			
Non ventilated/ventilated	24/26	NA	NA
Duration of MV (day), median (IQL)	5 (4–6)	NA	NA
PIM II score, median (IQL)	11.8 (2.4–33.7)	NA	NA
Fractional shortening (%), median (IQL)	28 (26–34)	38 (34–41)	<0.05*
cTnT (pg/mL), median (IQL)	22 (18–28)	10 (10-10)	<0.05*
Outcome, *n*			
Survivors/nonsurvivors	34/16	NA	NA

IQR: interquartile range; NA: not available; MV: mechanical ventilation; PIM: pediatric index of mortality; cTnT: cardiac troponin T; *significant.

**Table 2:** Correlation between levels of cTnT and various parameters in the critically ill children.

Variable	*r*	*P* value
PIM II score	0.670	<0.05*
Duration of ventilation (days)	0.691	<0.05*
Fractional shortening (%)	−0.600	<0.05*

PIM: pediatric index of mortality; *significant.

**Table 3:** Relation between levels of cTnT and outcome in the critically ill children.

Variable	Survivors (*n* = 36)	Nonsurvivors (*n* = 25)	P value
cTnT (pg/mL), median (IQL)	20 (18–24)	34.5 (27.5–41.5)	<0.05*

cTnT: cardiac troponin T; *significant.

## Abbreviations

CTnT: Cardiac troponin T
PIM II: Pediatric index of mortality II
IQL: Interquartile range
PICU: Pediatric intensive care unit.
